# Perceptions among diabetic patients in the ultra-orthodox Jewish community regarding medication adherence: a qualitative study

**DOI:** 10.1186/s12889-021-11619-6

**Published:** 2021-08-17

**Authors:** Inbar Levkovich, David Rodin, Shiri Shinan-Altman, Mordechai Alperin, Hodaya Stein

**Affiliations:** 1 Faculty of Graduate Studies, Oranim Academic College of Education, Haifa, Israel; 2 Department of Family Medicine, The Ruth & Bruce Rappaport Faculty of Medicine, Technion-Israel Institute of Technology, Clalit Health Services, Haifa and Western Galilee District, 36006 Kiryat Tivon, Israel; 3 Louis and Gabi Weisfeld School of Social Work, Bar Ilan University, 52900 Ramat-Gan, Israel

**Keywords:** Diabetes, Ultra-orthodox, Religion, Family physicians, Qualitative, General practitioner

## Abstract

**Background:**

Promoting a healthy lifestyle and achieving strict adherence to medical treatment among patients with diabetes are key objectives in public health. Yet health behaviors are often culturally driven, especially in closed religious communities. This study seeks to reveal key cultural-religious factors, attitudes and behaviors characterizing the lifestyle in one such closed community—the ultra-Orthodox Jewish community—by understanding the attitudes of ultra-Orthodox patients with diabetes toward coping with their illness and the factors impacting their adherence to medicinal treatment.

**Method:**

Qualitative interviews were conducted with 16 ultra-Orthodox patients with diabetes using a semi-structured, in-depth questionnaire.

**Results:**

Three main themes emerged: 1) “The disease as a secret”: Hiding the disease among patients with diabetes in ultra-Orthodox society; 2) “Distinguishing between sacred and secular occasions”: ultra-Orthodox diabetes patients distinguish between treatment adherence on weekdays and treatment adherence on holidays or special occasions; 3) “Ask the rabbi”: In cases of dilemmas that involved conflicts between halakhic rulings and doctors’ instructions, the rabbi’s decision was usually the final one.

**Conclusions:**

The findings of this study may help provide an in-depth understanding of the obstacles and motives of ultra-Orthodox patients in adhering to medicinal treatment of diabetes in particular and to medicinal treatment in general, thus helping family physicians who treat this population provide optimal and appropriate treatment.

**Supplementary Information:**

The online version contains supplementary material available at 10.1186/s12889-021-11619-6.

## Background

Diabetes is defined as a state of hyperglycemia in either fasting or postprandial states. The chronic hyperglycemia of diabetes mellitus (DM) is associated with a wide range of organ damage, dysfunction, and failure. Diabetes is one of the chief global health emergencies of the twenty-first century and is among the top ten causes of death worldwide. The International Diabetes Federation (IDF) estimates the overall prevalence of diabetes mellitus at 463 million in 2019, and this figure is expected to rise to 700 million by 2045 [1].

Glycemic control, which plays a main role in the management of diabetes, depends mainly upon patient adherence to the treatment plan. Indeed, accurate assessment of medication adherence is necessary for effective management of diabetes [2, 3]. Yet the medical profession has become increasingly aware that patients do not adhere to many aspects of health care advice. Previous studies have found that adherence to diabetes treatment is generally sub-optimal and ranges from 23 to 77% [4, 5]. The literature cites a number of factors related to health services and medicines, among them poor patient-provider relationships, time elapsed since diabetes education, medication side effects and pill burden [6–8]. In addition, different societies are known to have different health behaviors and different factors associated with rates of adherence to medical treatment [9–13].

Knowledge, attitudes and practices directly influence health behaviors and are often culturally driven. Indeed, culture affects almost all aspects of health care, including awareness of risk factors, early signs of disease, preventive medicine, diagnosis, health information and services [14, 15].

The Jewish population in Israel can be divided into different subcultures associated with diverse religious practices: ultra-orthodox *(haredi)*, orthodox *(dati)*, traditional *(masorti)* and secular *(hiloni)* groups. The ultra-orthodox group is characterized by stringency and conservatism in religious observance [16]. Jews affiliated with the ultra-orthodox group live in tightly knit communities marked by self-imposed cultural insularity [17]. These communities are characterized by orthopraxy in that they adhere to thousands of detailed commandments in all aspects of life, as dictated by the laws of the Torah [18]. Ultra-orthodox Jews tend to have high fertility rates based upon cultural practices anchored in the historical experience of the Jewish people [19, 20]. The worldwide population of ultra-orthodox Jews is currently estimated at 1.5 million, and due to their low rate of intermarriage and high birthrates, their numbers are growing rapidly. Ultra-orthodox communities are concentrated in Israel, the USA, and Western Europe [17, 21].

The social structure of ultra-orthodox communities is extremely closed and characterized by a hierarchical social system bound by religious values [22]. The communities are led by the rabbis and depending on the exact community the rabbis assume an important role in the lives of community members. Most ultra-orthodox Jews have little interaction with non-orthodox society [22].

The prevalence of diabetes among ultra-Orthodox patients is 15–18%, twice that of the general population in Israel [23]. To the best of our knowledge, no studies have examined diabetes patients within the ultra-orthodox Jewish community. Several studies have examined health behaviors among ultra-orthodox Jews. A study that compared ultra-orthodox Jews with the general population found that ultra-orthodox women were less likely to consume five portions of fruits and vegetables per day, more likely to consume more than five cups of sweetened beverages a week, and more likely to sleep less than seven hours a night than the general population. Obesity was also higher among ultra-orthodox women [24]. A retrospective cross-sectional study that examined ultra-orthodox people with diabetes and those without diabetes who visited the emergency room following the commencement of the Yom Kippur fast showed that the fast did not increase the rate of ER visits among adult patients with diabetes [25].

Religious belief also has critical repercussions on treatment, such that belief or faith in God is liable to develop into belief or faith in treatments. According to Rosmarin et al. [26], belief in God is associated with greater treatment credibility (patient perceptions regarding dependability and rationality of treatment) and greater treatment expectancy (patient expectations regarding treatment benefits). Treatment expectancy and credibility may improve the therapeutic alliance, as well as patient motivation and compliance with treatment [27, 28]. Recent studies have described the complex relationship of spirituality and religion to health [29, 30]. In a study examining the role of religion and belief among ultra-orthodox Israeli Jews hospitalized with a psychotic disorder, patients judged religion to be essential in the management of their illness [30]. Religious beliefs and prayer may improve one’s sense of control over stressful events and also instill a sense of purpose and meaning into events that are apparently meaningless, thereby strengthening one’s sense of belonging to the whole and to the universe [31, 32]. Understanding how the religious beliefs and health behaviors of ultra-orthodox patients may influence their disease and its treatment can have an impact on health behaviors such as medication adherence [18].

A growing body of literature is attempting to understand the special medical needs of the ultra-orthodox Jewish population [24, 27, 30]. Empirical data suggest that adherence to clinical recommendations for treating diabetes is inadequate in primary care and that a large proportion of patients with diabetes remain at high risk [33]. Consequently, a wide range of interventions have been implemented that are aimed at improving diabetes care and achieving better metabolic control for people with diabetes [34]. Family physicians play a significant role in treating these patients. The goal of this qualitative study was to examine attitudes, religious beliefs and health behaviors among ultra-orthodox people with diabetes and to understand their views and feelings about adherence to diabetes medication.

## Methods

The study used a qualitative-phenomenological approach [35]. This approach aims to obtain deep understanding of people’s behaviors and experiences by means of in-depth, semi-structured interviews. As our primary objective was to understand medication adherence from the patients’ perspective, we adopted this methodological approach, which allows flexible exploration of respondents’ experience and attitudes [35]. The descriptive power of this approach enabled us to achieve an in-depth understanding of how ultra-orthodox patients with type II diabetes perceive their disease, how it is treated and how they adhere to this treatment.

### Sample and population

This qualitative study was supported by a grant from the Israel Medical Association. The sample included 16 patients recruited from three primary care clinics affiliated with Clalit Health Services and located in the Haifa and Western Galilee District of Israel. These clinics were selected based on their high proportion of ultra-orthodox patients. Inclusion criteria for the study were individuals aged 18+, Hebrew speakers who define themselves as ultra-orthodox Jews and were diagnosed with type II diabetes mellitus by their family physician, who prescribed medication to control their diabetes. Based on the purposeful sample approach, we contacted participants who best represented the population and its heterogeneity in terms of age, education, and familial and socioeconomic status and who could best teach us about the studied phenomenon.

Family physicians at the three target clinics provided lists of patients diagnosed with diabetes. The names of patients who are not ultra-orthodox were removed from these lists. After that, the family physicians asked patients who met the inclusion criteria if they would be willing to receive further explanation from the researchers. The family physicians then gave the details of these consenting patients to two residents specializing in family medicine who had been trained in qualitative interviewing. These residents contacted the patients by telephone and explained the study’s purpose in detail. Patients were offered the opportunity to participate in the study and signed informed consent forms. They were told they could withdraw from the study at any time and refuse to answer any question. No incentives were offered. None of the candidates who received the explanation declined to participate in the research.

### Instruments and procedure

The study was approved by the Human Research Ethics Committee of Institutional Review Board of the Haifa and Western Galilee District of Clalit Health Services, the largest HMO (Health Maintenance Organization) in Israel, Approval No. 0161–18-COM1, and conducted in accordance with the Declaration of Helsinki. Informed consent was obtained in Hebrew. The researchers recruited the participants with the assistance of their primary care physicians.

The data were collected in 2020–2021 by means of in-depth, semi-structured interviews. We designed the interview guidelines with questions that would allow participants to share their experiences openly [36]. The interview guidelines included questions on several topics: health and disease perception, religious observance and acceptance of authority, role of the community in patient’s life. The interviewer encouraged patients to describe their experiences in their own words (Additional file 1).

The interviews were conducted by telephone. Prior to the interviews, the interviewees were asked to describe their religious status. All participants identified themselves as ultra-orthodox Jews. All the interviews were conducted in Hebrew and lasted approximately one hour. Data collection was completed when theoretical saturation was reached (i.e., additional interviews yielded no new material for analysis). The interviews were recorded and later transcribed verbatim. The interviews were conducted by telephone to comply with the social distancing guidelines for COVID-19.

### Data analysis

We used content analysis in this study. Where appropriate, we paraphrased and generalized the respondents’ statements. We then organized similar passages according to topic in order to identify individual influential factors, and then combined similar factors into major categories to identify the main themes. The researchers read all the data several times to achieve immersion and obtain a sense of the text as a whole. Then, the researchers read the transcript of each interview line by line, jotting down notes to identify initial units of meaning emerging from the data. The researchers reviewed the larger themes and discussed them. The researchers gradually detected context and content-related associations between sub-themes. Next, the researchers examined the interrelationships among the sub-themes order to theoretical themes. In the last stage, we identified and integrated the study’s central themes [36].

## Results

The 16 participants were between the ages of 53 and 74 (M = 64.37, SD = 6.11). The majority were born in Israel; 62.25% were men and 37.75% were women. Most were married (87.5%), and two were widowed (12.5%). Participants had between 10 and 17 years of education (M = 13.56, SD = 1.63) and an average of six children (range 1–14, SD = 2.84). Ten of the participants with diabetes had other family member with diabetes (62.25%). Most of the participants did not smoke (87.5%) and most checked whether their medications are kosher (62.25%). With respect to frequency of regular physical exercise, ten did not engage in any form of physical exercise (62.5%), three exercised once a week or less (18.75%), two exercised three times a week (12.5%) and one exercised more than four times a week (6.25%). Participants took the following medications for diabetes: Metformin (62.5%), Repaglinide (31.25%), DPP-4 inhibitor (31.25%), Sulfonylurea (18.75%), GLP -1 (6.25%), SGLT2i (6.25%), insulin (6.25%). Some of the patients take several medications, so that the total is over 100%). Total pill burden was defined as the total number of pills a participant took per day (range 0–5, M = 3.1, SD = 1.95).

Three central themes emerged from the quantitative analysis of the interviews (Table 1).

**Table 1:** Classification of main categories and subcategories

Main categories	Subcategories
“The disease as a secret”: Concealing the disease among patients with diabetes in ultra-orthodox society	• Most patients never discuss their illness with relatives or members of the ultra-orthodox community • These patients felt that diabetes is a personal and private matter • They fear that if their disease became known people might describe the situation as worse than it is • Patients whose parents have diabetes also described a similar pattern in which they acknowledge their disease but do not discuss it • Their belief gives them strength and serves as a meaningful coping resource
“Between the sacred and the profane”: Weekdays are different from holidays and special events in how ultra-orthodox people with diabetes perceive treatment adherence	• During the week they adhere to the guidelines, eat a healthy diet and avoid sweets • Things are different on Shabbat, when the extended family gathers for extravagant meals that include a variety of sweets • Special Shabbat delicacies are offered to them at the synagogue after the prayer service. Nothing suitable is available for people with diabetes • The participants also described their problems in maintaining a healthy diet on Jewish holidays • Benevolent organizations also are unaware of the need to provide food suitable for people with diabetes
“The rabbi is the address”: In dilemmas between religious law and doctors’ instructions, the rabbi decides	• GP plays an important role for them, yet the rabbis have the final word on any question that may arise • Patients in ultra-orthodox society believe that sickness and health are in God’s hands. • The patients see the rabbis as the highest source of knowledge about the Torah. Even if the rabbis are not knowledgeable about a particular medical issue, they can provide comfort, encouragement and answers • Measuring sugar levels and injecting insulin on Shabbat are not permitted, even if approved by the GP • Especially on Yom Kippur, fasting, constant prayer and avoidance of food and drink are of major importance.

### Theme 1: “The disease as a secret”: concealing the disease among patients with diabetes in ultra-orthodox society

People with diabetes in ultra-orthodox society indicated that while they had been living with their diabetes for years, most never discussed their illness with relatives, friends or members of the ultra-orthodox community, with the exception of their spouses and their primary care physicians. These patients felt that the topic of sickness is a personal and private matter that comes under their right to privacy. According to these patients, in the small ultra-orthodox community in which they live, rumors spread like wildfire. They expressed fears that if their disease became known they would have no control over who would find out and people might describe the situation as worse than it is. Therefore, they preferred to conceal or play down their medical condition so as not to give people a reason to talk.

“It’s called the Bnei Brak Communications network (BBC) . . . each person adds stories or elaborates on them . . . there are people in every community, even the ultra-orthodox community, who like to talk. If someone at the yeshiva wants to publicize some bit of disinformation, all he has to do is to visit a friend at twelve midnight, tell him something and caution him not to tell anyone else, and by the morning everyone at the yeshiva will know about it” (Patient 2).

Other participants stated that they avoid talking about this subject even when they know the other person has a chronic disease or perhaps even diabetes. One participant told us that even though she knows her friend has diabetes; she will not reveal that she also has the disease for fear of how her friend will react. Among some of the participants, diabetes is perceived as a disease of lazy and fat people that should be played down and that people with diabetes are liable to become a burden to society. Patient 3 described how he avoids attending events that conflict with his scheduled medication times to prevent people from asking questions:

“For example, today I’ve been invited to a wedding in Bnei Brak. When there are other people present, should I take my pill in their presence or wait until I get home? I don’t need to be asked why I am taking a pill or what is wrong with me. It’s none of their business. People don’t need to know. These are personal, confidential, medical matters. No one needs to know about it” (Patient 3).

The patients also stated that they conceal their disease even from those closest to them—their grown children. Some said that their children know they take medication but don’t know why, while others said that they tend to hide their treatment. Some even avoided saying the word “diabetes” and said “the disease” instead. They generally felt that if their children had not developed diabetes, they had succeeded in protecting them and did not want their support.

“My wife knows and makes sure I eat the right food. Maybe my children know. I’m not sure. I haven’t discussed this with them . . . I didn’t tell them directly that I have this problem. It just never came up. I don’t know, you just don’t talk about these things . . . I know that my children are healthy, thank God. They don’t have any particular problems” (Patient 3).

Patients whose parents have diabetes also described a similar pattern in which they acknowledge their disease but do not discuss it. Expressing interest in or asking questions about the disease is perceived as invasive and voyeuristic behavior that is not appropriate in this community. Only the patient’s wife has a reason to be involved, mainly as the one responsible for cooking and nutrition in the home.

“I know it is hereditary, that’s what I know. In my father’s family, everyone had diabetes. . . I have no idea if my father’s diabetes is under control or not . . . I don’t know whether he takes medication. I do know that he tests his blood sugar but I don’t know if he takes medication . . . possibly he cheats here and there. He goes to weddings and the like. At home my mother is on guard and keeps him in line” (Patient 5).

As in the case of other diseases, diabetes is not discussed openly in the community, leaving patients to fend for themselves. Their belief gives them strength and serves as a meaningful resource: “Those who believe have nothing to fear.” Ultra-orthodox women play a major role in cooking for the family and preparing for the Sabbath and the many holidays. Hence, the findings show that women interviewees took responsibility for their own nutrition. Men, in contrast, left this responsibility in the hands of their wives, and most never discussed other aspects of their illness. Their main discussions on these aspects were with their primary care physicians, with whom they discussed adhering to their medical treatment, engaging in physical activity, not smoking and more. Within the community the matter of illness was minimized, leaving the family physician as the main address for such discussions:

“No, no, no. We do not talk about this in the community. It’s not something to talk about . . . it exists, but I have my doctor. I don’t focus on this too much, I don’t pay it too much attention. I know it’s there, I’m aware of my limitations, so I take care of myself more or less and that’s it” (Patient 8).

Marriages in ultra-orthodox society are usually arranged by a matchmaker and the parents, who make inquiries about the designated couple before the match is made. After the initial inquiry, the prospective bride and groom meet alone at a public place and decide whether to marry. The research participants were ambivalent about whether they should reveal their diabetes during the matchmaking process. Some acknowledged the need for transparency regarding the family’s medical history. Others claimed there is no need to reveal this disease for it has no major implications and is liable to spoil the match:

“He doesn’t need to hang out a sign but he does need to let them know. There’s some talk about people who conceal this, but the clear and unequivocal worldview of renowned Torah scholars is that this is forbidden, you must not play down something that is liable to be discovered later – this is a matter of agreement under false pretenses . . . but let’s say that not everything needs to be publicized” (Patient 2).

### Theme 2: “Between the sacred and the profane”: weekdays are different from holidays and special events in how ultra-orthodox people with diabetes perceive treatment adherence

Most of the research participants claimed that during the week they conform to the guidelines, eat a healthy diet and avoid sweets. One participant stated that she purchases fruit and popsicles for her more than 40 grandchildren but is never tempted to eat these herself. Nonetheless, the research participants admitted that things are different on the Sabbath, when the extended family gathers for extravagant meals that include a variety of sweets. Patients with diabetes who stated that they maintain good nutrition throughout the week described in detail how the Sabbath is different and how hard it is for them to maintain a proper diet and resist the temptations of *Oneg Shabbat* (the joy of the Sabbath meal):

“On Shabbat I do not maintain my diet. No way! . . . There is jahnun (Yemenite pastry), full of carbohydrates based on margarine, out of this world. I drink dietetic beverages, I eat fruit and nuts and seeds. So on Sundays when I check my sugar, it can be anywhere from 180 to 230. Afterwards it goes down to 120. . . I immediately drank half a glass of grape juice and two rugelach (sweet pastry) to balance things. There are always cookies and the kubana (Yemenite bread) my wife prepares for Shabbat . . . but I don’t eat kubana on weekdays, only on Shabbat morning” (Patient 3).

Some of the patients reported that the special Shabbat delicacies are offered to them at the synagogue after the prayer service. Nothing suitable is available for people with diabetes and they feel they are being discriminated against. Nevertheless, because no one knows about their disease, no fitting alternative is offered. Getting together after prayer services is a social and community obligation in which they feel they must participate. They explained that the temptation to eat something forbidden is an evil inclination.

“After prayers on Shabbat they serve noodle kugel—a ton of carbohydrates with sugar and potatoes, God help us, and herring and crackers and drinks. ‘Can you bring out a bottle of diet drink,’ I ask. The response is: ‘We don’t have any.’ ‘Why not?’ I ask. ‘There are people here who don’t drink sugared drinks. They don’t want to drink soda but they want to drink something’” (Patient 2).

The participants also described their problems in maintaining a healthy diet on Jewish holidays. They described the special dishes they love so much and what a hard time they have resisting them. Moreover, a few participants stated they made sure healthier substitutes were available. The major impression was of a dam that was breached on the Sabbath and on holidays and of personal and social difficulties in complying with the guidelines they managed to adhere to during the week:

“On Passover there are matzahs. The holiday is chock full of calories. I love matzahs, especially when my wife fries them with eggs, with a tomato . . . I’m crazy about that fried matzah, wow! I can devour as many pieces as she makes. There’s nothing else to eat, nothing. So that’s what I eat. I usually am very careful, very careful” (Patient 3).

The ultra-orthodox community is a closed community that engages in many charitable activities, particularly during crises. Some of these ongoing activities are benevolent activities characterized by doing good deeds for needy others by providing them with food, medicine, financial help and more. Some of the participants mentioned that these benevolent organizations also are unaware of the need to provide food suitable for people with diabetes. Most of the food in the care packages for the needy is rich in carbohydrates and sugars.

“Some of the gemachs (charitable societies) provide food. Food rather than money. Fruit, vegetables, meat, bread, and the like. They prepare special baskets for families, and I know that some families also receive money . . . There’s Rabbi XX, for example, he organizes this or adds money, I don’t know. There’s a fund that he collects and then donates the money…. People come, mainly before holidays, or if a family is having trouble, I know they collect money. Collect money and send it to them. Or they send food . . . and mainly carbohydrates because they are more filling, carbohydrates and sugars are more filling.” (Patient 11).

### Theme 3: “The rabbi is the address”: in dilemmas between religious law and doctors’ instructions, the rabbi decides

The research participants stated that in ultra-orthodox society, the rabbis are important personages and are the supreme authority when it comes to questions of religious law as well as to the way individuals conduct themselves in public and in society. All the research participants completely agreed that the family physician plays an important role for them, yet on any question that may arise it is the rabbis who have the final word. Patient 9 stated that whenever she is asked who is the most influential person for her, she answers that it is the community rabbi. “He is the soldier of God, the Creator of the Universe.”

“Since the days of Moshe Rabbenu (Moses our teacher), the rabbis have always been the leaders . . . the rabbi is the one to ask. Everyone, thank God the community has grown and everyone has his own rabbi . . . in general, the community asks and consults . . . If it is a critical matter and someone heard that there are medical differences of opinion he will go to the rabbi, or the rabbi will refer to him to one of the unbiased medical experts . . . in all such cases you must consult the rabbi” (Patient 2).

Patients in ultra-orthodox society believe that sickness and health are in God’s hands. The rabbis are experts in good deeds and in rules and they also maintain confidentiality. The patients see the rabbis as the highest source of knowledge about the Torah. Even if the rabbis are not knowledgeable about a particular medical issue, they can provide comfort, encouragement and an answer to any question that arises:

“Our Merciful Father, Blessed Be He, he is the one who gives the disease and also gives the cure. So you know that recovery will come and that the disease and its cure come from the same source. You don’t feel well, like someone who comes to the rabbi and says, ‘Your honor, a miracle happened to me, I fell from the sixth to the fourth floor and nothing happened to me. God must really love me.’ So the rabbi says to him, ‘Who dropped you? The one who loves you?’ You need to understand that there are no satanic acts, that everything is a matter of divine providence. The disease and, with God’s help, also the cure.” (Patient 9).

Patient 1 stated that she frequently consulted with rabbis about medical complications and explained that when you bring a dilemma to the rabbi, you must accept his decision as final:

“If the rabbi tells you not to have the surgery on a particular date or not to have surgery at all, you will see from the results that it was good that you asked. This has happened in many many cases, that people went to ask the rabbi and he told them not to do something and that helped, it was for the best. There are so many cases that if you ask, you must do what the rabbi says. If you ask, you cannot refuse to do what he says. You must accept what you are told” (Patient 1).

From the participants’ perspective, measuring sugar levels and injecting insulin on Shabbat are not permitted, even if approved by the general practitioner. According to Jewish law, pricking oneself on Shabbat is prohibited. Some of the participants stated they would not ask the rabbis about this, while others considered it to be “saving a life.”

“I check myself every day, every day I prick my finger, test my blood and that’s it. I do this to monitor my condition, so God forbid I do not get to a state where my sugar is too high or too low. Sometimes it is too low, I don’t know why. Sometimes it is too high. Every time I test myself, I record the results, I record everything . . . no, not on Shabbat. On Shabbat it is forbidden, forbidden to draw blood. No way will I do this on Shabbat” (Patient 16).

The matter of fasting was also brought up in all the interviews, and particularly the fast on Yom Kippur, a day that is especially holy and entails suffering, constant prayer and avoidance of food and drink.

“Even though the medical team told me not to fast, fasting was very important to me. My faith is extremely important to me, and I said ‘I’m fasting, even over my dead body.’ Even though the doctors warned me not to, I fasted” (Patient 10).

Moreover, when a dilemma concerning a medical matter is brought to the rabbi, whether their general practitioner is also ultra-orthodox is of major importance. The assumption is that a doctor who is not ultra-orthodox is likely not to understand the importance of the fast for a patient with diabetes.

“If the doctor does not follow the dictates of the Torah, the matter of the fast may not mean anything to him. So the rabbi asks who the doctor is” (Patient 3).

## Discussion

The goal of this study was to examine attitudes, religious beliefs and health behaviors among ultra-orthodox people with diabetes and to understand their perspectives and attitudes regarding adherence to medication for treating their diabetes. Overall, the findings of this study provide support for existing knowledge regarding the key role of religion in shaping attitudes, perceptions and health behaviors and underline the importance of paying attention to religious contexts to gain a more complete understanding of the lived experiences of religious populations [37].

The findings of the first theme paint a disturbing picture regarding how ultra-orthodox people with diabetes cope with their illness. Interviewees stated that they rarely discuss their illness with relatives, friends or other members of the ultra-orthodox community, such that in effect they are living with a secret. Keeping the disease a secret is a cause for concern for three main reasons. *First*, the fear of exposing the disease to close family members or social circles may lead to difficulties in adherence to medical treatment caused by fear of exposure. Since disease concealment was of major value to the ultra-orthodox patients, their medical decisions may have been influenced by this value. For example they may prefer oral medications over insulin. *Second*, keeping a disease as a secret can lead to negative emotional reactions such as depression and anxiety [38]. *Finally*, keeping a secret from the whole community can lead to distancing from community members and may generate ambivalent feelings about the sense of community belonging. These ambivalent feelings may be particularly complex in the ultra-orthodox community, where interpersonal relationships and a sense of belonging are highly important [39].

Participants appeared to fear being stigmatized, even though they did not mention this directly. Stigmas are defined as discriminatory behaviors directed toward people with the stigmatized condition [40]. Previous studies have shown that people with diabetes are stigmatized as sick and disabled [41]. Being stigmatized was also found to cause a greater burden for people with diabetes in certain population sub-groups, such as women and young adults [42]. This is especially important for ultra-orthodox people with diabetes, as a person’s lineage is an important component of social status. Low social status exerts a negative impact on arranged marriages, which are the norm in ultra-orthodox community, as parents want their children to marry into a family of the same social status or higher when arranging a marriage [43].

The findings of the second theme show that patients with diabetes generally adhere to the treatment of their disease. This finding is consistent with previous studies which found that faith and religion promote adherence to disease treatment [30, 44]. For example, a study conducted among 30 ultra-orthodox Jews with psychotic illnesses found that spirituality and religious practice provide meaning to their disease, empower them to combat their disease and help them not feel alone in confronting their illness because they have support from God or their community) [30]. Yet one interesting finding of the present study is that although the interviewees routinely adhere to their treatment, in some situations religious practices (e.g., eating festive meals on holidays) make it difficult to adhere to treatment. Overall, family food habits and personal food preferences are known to be serious challenges that made dietary adjustment difficult for people with diabetes [45]. Among ultra-orthodox patients with diabetes, this adjustment becomes especially challenging because not fully participating in religious practices is usually not an option for them [46]. These difficulties generate conflict between their desire to conform to religious commandments (mitzvot) and take part in festive meals and their guilt feelings about eating and not adhering to their treatment, thus impairing their health. Note, however, that a study that examined the effect of the Christian holiday season among patients with diabetes found that glycemia and lipids increased post-holiday, though the effect was small and transient [47].

As illustrated in theme three, the rabbi is a significant support figure in coping with the disease. The lives of ultra-orthodox people are mostly guided by the community leader, the rabbi or other Jewish spiritual leaders [22]. As in previous studies in which rabbis were found to be supportive and advisory figures (e.g., [43]), in the present study as well participants noted that even if the rabbi is not knowledgeable about a particular medical issue, he can provide comfort and can answer any question that arises. Moreover, when a dilemma concerning a medical issue is brought to the rabbi, whether the GP is also ultra-orthodox is of major importance. The assumption is that a doctor who is not ultra-orthodox is likely not to understand how important religious practices (e.g., fasting on some holidays) are to patients with diabetes. It seems that for some participants, religious practices such as conforming to mitzvot and consulting with the rabbi provided them psychological benefits that positively influence how they experience their illness. Accordingly, intervention programs should take positive religious coping mechanisms into consideration to improve the quality of life for people with diabetes [48].

All three themes in this article provide a clear picture of the intersection between basic elements of religious beliefs and the experience of coping with diabetes. On the one hand, religion serves as a resource for coping with the disease. As an internal resource, a sense of belonging to the community and the belief that sickness and health are in God’s hands provide a sense of relief and somewhat help participants cope with the disease. These findings are compatible with previous studies which found that positive religious coping and trust in God were strong correlates of less stress and increased positive impact [49]. On the other hand, the interviewees experienced feelings of concealment and isolation as well as guilt if their disease was revealed. Thus, they are conditionally allowed to enjoy the benefits of the community as long as they keep their disease as a secret and cope with the concrete and emotional consequences of keeping the disease as a secret.

### Limitations

This study has a number of limitations. First, it only examined the perceptions and experiences of patients being treated at public clinics and may not be a representative group. Thus, the results may not be applicable to patients with diabetes who avoid treatment. Nevertheless, this study included patients with varied backgrounds in terms of illness and treatment characteristics and varied socio-demographic backgrounds, facilitating a diverse view on their perceptions and experiences. Second, due to the nature of qualitative research, the sample size in this study was limited. Third, this study only examined patients treated by ultra-orthodox physicians. Future research should examine treatment compliance when the family doctor is from a different cultural group. Moreover, as a result of the COVID-19 outbreak, we were unable to interview the participants face-to-face and were forced to collect the data via telephone interviews, as members of the ultra-orthodox community do not use the Zoom platform. In the future, research should examine the perceptions of patients with diabetes in the ultra-orthodox Jewish community over time.

### Implications

This study has several practical implications. The challenges faced by ultra-orthodox patients with diabetes also serve as opportunities for clinicians to practice sensitivity, gain insight and provide quality care through a process of inquiry and consideration of the unique characteristics of this community. Given that Israeli healthcare providers do not receive any kind of training about ultra-orthodox Jewish culture in medical schools, we recommend that such special training be included in the medical school curriculum. A deeper understanding of the specific cultural context of the experiences of people with diabetes is important for designing effective cultural psychosocial interventions, as well as sensitive physician–patient communication [50]. Personal and group interventions should be tailored to the unique religious background of these people but should also consider the social norms of the lay community. For example, on the societal level, stigmas and fears related to diabetes among the lay public should be reduced to help patients cope with the disease. In addition, patient outcomes for obese patients can be improved if physicians gain familiarity with some of the unique religious, cultural and social characteristics of these patients. Health providers should adopt therapeutic approaches based on cultural competence that take into account patients’ beliefs, values and norms. Lastly, physicians are advised to collaborate with the rabbis of their patients with diabetes to determine how to promote treatment that is consistent with religious beliefs and practices.

## Conclusions

This study has provided a comprehensive and in-depth understanding of ultra-orthodox patients with diabetes in Israel. In this community, the rabbi is a significant support figure in coping with the disease. Ultra-orthodox people with diabetes rarely discuss their illness with relatives, friends or family members, such that they live with a secret and fear being stigmatized. In addition, the Sabbath and holidays are special occasions in how ultra-orthodox patients with diabetes perceive treatment adherence.

The current study provides practical recommendations for GPs who treat people with diabetes in the ultra-orthodox community. Taking into consideration the religious, cultural and social characteristics of patients with diabetes can promote better patient outcomes and improve physician-patient communication. Group interventions led by GPs from the ultra-orthodox community or in conjunction with rabbis are likely to help in treatment adherence.

## Supplementary Information

**Additional file 1.** Interview questions posed to diabetic patients in the ultra-Orthodox Jewish community.

## Data Availability

The datasets used and/or analysed during the current study are available from the corresponding author on reasonable request.
