# Supplementary material for: Perceptions among diabetic patients in the ultra-orthodox Jewish community regarding medication adherence: a qualitative study
Source: BMC Public Health. 2021 Aug 17;21:1559. doi: 10.1186/s12889-021-11619-6 (PMC8369440; doi:10.1186/s12889-021-11619-6)
Supplement: Supplementary file 1 — Additional file 1. Interview questions posed to diabetic patients in the ultra-Orthodox Jewish community. [file 12889_2021_11619_MOESM1_ESM.docx]

**Appendix 1. Interview questions posed to** **diabetic patients in the ultra-Orthodox Jewish community**

**Health and disease perception**

- How do you view your general health? your diabetes?
- What in your life helps you maintain your health? What helps you improve your health?
- Do you have any fears about your health in the present and the future? If so, can you tell me about them?
- How does your family relate to your diabetes? How do you cope with your diabetes in your everyday life?

**Adherence to treatment for diabetes**

- How would you describe your compliance with medications for treating your diabetes? your compliance with medications for treating other diseases?
- Describe any difficulties you experience in complying with your medications in treating diabetes.
- If you could change anything in the medications you take or the directions for taking them to achieve maximal treatment compliance, what would you change?

**Religious observance and acceptance of authority**

- Do you consult any authority figure on a regular basis about matters of Jewish law?
- Can you recall any incident where you had difficulties deciding between your diabetes and your religious beliefs? Tell me about it. Who did you turn to for advice? What did you do?

**Role of the community in patient's life**

- What is the role of the community in your daily routine? in your life cycle in general?
- To what extent does the community influence your decisions in various areas of your life?
- Have you told any members of your community about your diabetes? If so, what was their response?
- What do you believe is the prevalent opinion in your community about diabetes? What is known about the disease?

**Relationship with your GP**

- How do you see your relationship with your GP?
- Do you believe that your GP’s religious background influences your relationship?
- What recommendations can you make to other people with diabetes in ultra-orthodox society?
